# “Show this thread”: policing, disruption and mobilisation through Twitter. An analysis of UK law enforcement tweeting practices during the Covid-19 pandemic

**DOI:** 10.1186/s40163-020-00129-2

**Published:** 2020-10-21

**Authors:** Manja Nikolovska, Shane D. Johnson, Paul Ekblom

**Affiliations:** Dawes Centre for Future Crime at UCL, University College London, London, UK

**Keywords:** Crime reduction, Crime prevention, Police, Twitter, Covid-19, Disruption, Crisis communication, Evidence-based policing, Social media policy

## Abstract

Crisis and disruption are often unpredictable and can create opportunities for crime. During such times, policing may also need to meet additional challenges to handle the disruption. The use of social media by officials can be essential for crisis mitigation and crime reduction. In this paper, we study the use of Twitter for crime mitigation and reduction by UK police (and associated) agencies in the early stages of the Covid-19 pandemic. Our findings suggest that whilst most of the tweets from our sample concerned issues that were not specifically about crime, especially during the first stages of the pandemic, there was a significant increase in tweets about fraud, cybercrime and domestic abuse. There was also an increase in retweeting activity as opposed to the creation of original messages. Moreover, in terms of the impact of tweets, as measured by the rate at which they are retweeted, followers were more likely to ‘spread the word’ when the tweet was content-rich (discussed a crime specific matter and contained media), and account holders were themselves more active on Twitter. Considering the changing world we live in, criminal opportunity is likely to evolve. To help mitigate this, policy makers and researchers should consider more systematic approaches to developing social media communication strategies for the purpose of crime mitigation and reduction during disruption and change more generally. We suggest a framework for so doing.

## Introduction

The Covid-19 pandemic has had a profound effect on society worldwide, influencing how we work, interact with others, and travel. Unsurprisingly, it has also had an impact on crime, with studies suggesting that lockdown restrictions have been associated with reductions in crimes reported to the police for offences including burglary (e.g. Ashby 2020; Halford et al. 2020; Felson et al. 2020), shoplifting (e.g. Halford et al. 2020), and assault (e.g. Halford et al. 2020). Studies concerned with domestic abuse (Usher et al. 2020, Piquero et. al 2020; Campbell 2020; Chandanet et al. 2020; Boserup et al 2020, Pfitzner et al. 2020) have produced mixed results, with initial spikes being followed by reductions in calls for police service. With such studies it is unclear whether the reductions observed represent reductions in offending or the rate at which offences are reported to the police. Regardless, the patterns observed suggest an impact of the lockdown on these types of crime. While increases in crime have also been reported for cybercrime (Buil-Gil 2020; Hakak et al. 2020), including online fraud (e.g. Naidoo 2020; Cimpanu 2020), malware (Brumfield 2020), hacking and phishing (Muncaster 2020; Kumaran and Lugani 2020), Hawdon, Parti and Dearden (2020) report that cybercrime remained unchanged despite the swift change in routine activities. However, data on such crimes is more elusive and analyses—at least in the academic and open source literature—less complete than for more traditional crimes such as those discussed above.

Interestingly, previous research on the impact on crime of previous epidemics/pandemics is limited. Research (Fong and Chang 2011) conducted during the 2003 SARS epidemic examined community collective efficacy in Taiwan in communities that experienced SARS and those that did not. However, the authors did not directly examine the effect of SARS on crime. For this and other reasons, understanding the extent to which Covid-19 has impacted crime is important and will doubtless feature strongly in academic research in future.^1^

To provide a complete picture of what has and will happen, will require access to police recorded crime data, but also that reported to, or collected by other organisations. This is because not all crimes (e.g. domestic abuse) are reported to the police and because patterns of reporting may have changed during the lockdown. Additional insight may also be gained about patterns of offending, and concerns about this, from analysis of data posted to social media platforms, such as Twitter. In this paper, we analyse data from UK government and law enforcement Twitter accounts with a view to understanding how law enforcement used this platform to inform the public about crime risk and what to do about it during the early stages of the pandemic. While our focus here is on the Covid-19 pandemic, we consider this to be just one example of disruptions to society with the potential to impact on crime opportunity and motivation, and security. As such, we view the research that follows as having implications for other future large-scale disruptions, and national and global emergencies, and how society prepares for them, including anticipating their consequences for crime and security.

### The web of Police influence

The use of social media to communicate in times of crisis or disaster has become essential for the mitigation, coordination and recovery of societies hit by disruptions (Houston et al. 2015). For example, to deal with security and public safety during the pandemic, law enforcement and government agencies cannot act alone. A web of influence has to spread out from these (and other) stakeholders to (for example) other agencies, private businesses and householders to enable them to play their part in mitigating both the pandemic and its knock-on effects on other aspects of life, such as crime and security. Characterising that web and how it works is vital to targeting, assessing and improving the influence process. In the UK, ‘communication policing’, or open source police communications that use the internet and social media, has been characterised as a new form of community policing by the. Open Source Communications Analytics Research [OSCAR] Development Centre.^2^ Their research on the use of open source communications by police suggests that social media communications should be routinely incorporated into police investigations, intelligence gathering and community engagement. In the present study, we focus on how UK law enforcement institutions have sought to communicate with and influence others to undertake, or desist from, a range of actions as required. In setting up the paper, we first discuss existing crime science approaches for describing and assessing how ‘professional security influence’ is spread. There will be many useful parallels in other policy areas, such as medicine (e.g. see Michie et al. 2011) and generic influence processes such as the ‘nudge’ approach (Halpern 2015), but our focus here is more limited.

In studying the dissemination of influences on people’s behaviour, and that of organisations, it is helpful to think about roles to be played, and associated with these, the accompanying responsibilities. Opportunity theories of crime (e.g. Cohen and Felson 1979) note that crime can only occur when a likely offender and victim converge at a particular place (on or offline) and time, absent a capable guardian. However, these are clearly not the only actors involved. The likelihood that such convergences will occur, and whether they are conducive to crime, is further influenced by the actions of place managers, and ‘handlers’. Handlers are those who have an emotional attachment to a particular offender (e.g. parents, friends) and can exert some control over them (e.g. discouraging them from offending). Place managers on the other hand are directly responsible for specific locations (e.g. shops, bars, hospitals), and can (for example) ensure the environment is designed to make crime more difficult (e.g. by placing expensive items behind a counter in shops), by training their staff to act in particular ways, or by employing specific tactics that deter crime or de-escalate situations as they arise. Extending the conceptual framework further, Sampson et al. (2010) note that the actions of guardians, place managers and handlers are influenced by ‘supercontrollers’, who can include formal organisations (e.g. regulators, government departments, police forces), diffuse collectives (e.g. the media), as well as more personal networks (e.g. families). While guardians, place managers and handlers can have a direct influence on the likelihood of a crime event taking place, supercontrollers exert their influence indirectly via the impact they have on these latter ‘controllers’.

Other approaches are also relevant. Mazerolle and Ransley (2005) introduced the concept of ‘third-party policing’, describing a blurring of the boundary between law enforcement and civil action to tackle crime. To all these ‘crime preventer’ roles, Ekblom (2011) adds the concept of ‘crime promoters’—people or organisations that, inadvertently or deliberately, increase the risk of crime, and hence who must be influenced to desist. He also introduces the concept of involvement as a separate crime prevention task from the practical side of implementation, centering on the actions of alerting, informing, motivating, empowering and directing individuals and organisations to undertake particular crime prevention roles/responsibilities that have been identified and assigned. Both these additions will be returned to in the discussion section of the paper, but for now it is important to note that Twitter can be used as a medium to encourage (or discourage, as appropriate) individuals or those with a responsibility to reduce crime, to act.

In what follows, we examine how UK law enforcement used Twitter during the early stages of the pandemic to alert the public and others about crime problems, inform them about how they are committed, and to empower them to reduce their risk, or the actions they could take if victimised. In the context of crime prevention, much has been learned about what works to reduce crime (e.g., Weisburd et al. 2016). However, as far as we are aware, the evidence base regarding police use of social media to involve people and other agencies in implementing or supporting security interventions is under-developed (see below). As such, this study represents an attempt to catalyse activity in this area. While it is out of scope to examine if law enforcement use of Twitter actually influenced the behaviour of the stakeholders listed above (including potential victims), we examine the following related questions: what is tweeted; whether messages are sufficiently retweeted for them to have the potential to have their desired effect; what factors, if any, are associated with whether or how frequently messages are retweeted; and whether messages provide advice that empowers citizens (or others) to act? In the next section, we briefly review research concerned with Twitter use and the pandemic, before presenting our methodology and results.

### Twitter, law enforcement and disruption

In 2019, the micro-blogging platform Twitter reported 320 million active users and over 500 million daily posts. As of July 2020, Statista reports that the UK ranks fifth in terms of Twitter active users, with just over 15 million. The popularity of the platform and its use has consequently attracted much data-driven research (Miró-Llinares et al. 2018; Ashktorab et al. 2014; see also: Cheong and Lee 2011; Kumar et al. 2011; Mandel et al. 2012; Imran et al. 2013). While the general public’s engagement with the platform has raised its popularity, public bodies and government agencies across the world commonly employ Twitter to communicate with the populace, via their own verified user accounts. Previous research (Crump 2011; Lee and McGovern 2013; Heverin and Zach 2010; Lieberman et al. 2013; Walsh 2019) has shown that law enforcement agencies (LEAs) may use Twitter and other social media platforms for operational purposes (e.g. sharing alerts, warnings, up-to-date and verified information); for building community trust, involving and educating citizens in and on the governance of crime, risk and insecurity; and for sharing successful enforcement stories. In relation to this, the use of Twitter by LEAs has been saluted for enhancing “police-citizen encounters and the foundational goals of community policing—fostering non-adversarial relations through public participation, decentralised decision-making, and two-way communications” (Walsh 2019:3). The use of Twitter by LEAs has also been found to increase transparency, which can (to some extent) increase police legitimacy (Grimmelikhuijsen and Meijer 2015).

In the UK, LEAs started to use social media around 2008, with North Yorkshire and West Midlands police taking the lead by using Facebook and YouTube to share information about local policing (Crump 2011). It was anticipated that Twitter would not become the main platform from police-citizen engagement as it was difficult for general users to engage with Twitter discussions (Heverin and Zach 2010; Crump 2011; Lieberman et al. 2013); but the platform has since evolved. The Twitter of today has become a primary platform for the sharing of (media-rich) information and news during crises. Denef et al. (2013) studied the tweeting practices of two UK police forces during the August 2011 riots, finding that one adopted a more formal, or depersonalized approach, while the other adopted a highly personalized, informal and interactive style which also included interaction with users. They conclude that, as different communication strategies may influence public engagement with police content on social media, there is a need to adjust communication strategies and polices to the local context (see also: Meijer and Thaens 2013). Police tweeting practices have now become popular, but Dekker et al. (2020) suggest that police social media policies inadequately address the barriers, structural and cultural, that may arise—and will need to adapt. They note the benefits of user engagement that Twitter affords, including learning from the public.

While research on police use of Twitter has received relatively limited attention (particularly in times of crisis, and in terms of the approach taken in this paper), research on Twitter use during epidemics has received substantially more, mostly focusing on changes to public awareness and the reporting and spread of outbreaks (Broniatowski et al. 2013; Grover and Aujla 2015; Ji et al. 2012; Smith et al. 2015; Diaz-Aviles and Stewart 2012). The swine flu outbreak in 2009/10 was the last and most recent pandemic that attracted Twitter-driven research. Most of this research examined public perceptions, or involved the gathering and analysis of Twitter data regarding the sharing of information about that pandemic (Ahmed et al. 2019; Chew and Eysenbach 2010; Kostkova et al. 2014; McNeill et al. 2016; Ritterman et al. 2009; Signorini et al. 2011).

Unsurprisingly, research on the Covid-19 pandemic using Twitter data is gathering pace. For example, Cinelli et al. (2020) used Twitter and other social media data to examine the diffusion of information regarding Covid-19 for the period 1 January to 14 February 2020. Alshaabi et al. (2020) analysed the spread of the use of the word ‘virus’ among languages to track how the Covid-19 pandemic has been discussed through late March 2020 on Twitter. Further, in their study, Dong et al. (2020) created an interactive web-based dashboard that tracks Covid-19 in real time using Twitter feeds, while Chen et al. (2020) have created the first public coronavirus Twitter dataset (which is continuously updated).

However, to the best of our knowledge to date, Twitter data has not been used to examine the Covid-19-crime association, or law enforcement use of Twitter during the pandemic. For the purposes of this study, to answer the research questions outlined above, we concentrate on user-timeline Twitter data concerned with Covid-19 from public sector stakeholders involved in crime reduction across the UK. In what follows, we first describe the approach taken to sampling and data collection. Next, we discuss our analytic approach and present our findings. We conclude the article with a discussion of our findings, what they might mean for policy and practice, and future research directions.

## Method

### Data collection

We first identified each of the police forces (territorial and national) in England, Wales, Scotland and Northern Ireland, along with the other UK agencies with responsibilities for crime reduction (e.g. the Home Office, National Police Chiefs’ Council, the College of Policing, Action Fraud^3^ and Neighbourhood Watch). The full list of (75) stakeholders considered in this study can be found in Additional file 1: Stakeholder list. Next, we manually searched for the primary verified Twitter accounts for each of the stakeholders. We opted to analyse the activity of only the primary accounts for each stakeholder as—while other accounts exist^4^—we reasoned that these would be the accounts that the general public typically engaged with. Moreover, there currently exists no comprehensive repository off police twitter accounts, which makes the systematic identification of other accounts difficult (for us and the general public). On 23 May 2020, the R package ‘rtweet^5^‘ (Kearney et al. 2019), was used to download the tweets posted and retweeted by these accounts. While we could not collect tweets for a specified period, the ‘user_timeline’ search function enabled us to download the previous 3200 tweets published by each stakeholder (up to the collection date). This resulted in the extraction of 236,609 tweets from all stakeholders. Due to differences in the frequency with which stakeholders posted tweets, the date of the first tweet varied for each stakeholder. However, complete data were available for all stakeholders from 1 September 2019. As such, we analyse trends in the data from this period to 10 May 2020, which was the date on which the UK Government published its plans for the easing of the lockdown and changed its messaging from ‘Stay Home’ to ‘Stay Alert’. This equated to a total of 114,257 tweets. In selecting the data for this period, this enabled us to analyse Twitter data for the 5 months prior to and since the onset of the Covid-19 pandemic. In future work, we aim to analyse data for later intervals. For each tweet, we downloaded data for 90 variables including the name of the Twitter account, the date and time of the tweet, the text tweeted, and the number of likes and times the tweet was retweeted.

### Analytic strategy

Automated approaches have been developed for the purposes of extracting and analysing large volumes of text data. These include sentiment analysis (Pak and Paroubek 2010; Kouloumpis et al. 2011) and message polarity (Lima et al. 2015). However, the reliance on such approaches has been criticized for missing the deeper context or meaning of communications (Walsh 2019). This is particularly likely to apply to novel datasets for which such techniques may not work well. Law-enforcement tweets may include information on various sorts of crime, the publicising of policing actions, as well as interactive content and suggested crime prevention advice (Walsh 2019). Moreover, when we consider the novelty and disruption to social settings that Covid-19 has engendered, such information can become inconsistent and highly variable. For example, many law enforcement agencies have been committed to raising awareness of social distancing and the policing of Covid-19 restrictions.

For these reasons, our initial analytic strategy involved the use of a qualitative approach, in this case a thematic analysis (see, Strauss and Corbin 1998; Walsh 2019; Heverin and Zach 2010; Crump 2011; Lieberman et al. 2013). This allowed us to immerse ourselves in the data and capture the richness of its content. Our approach to coding is discussed next.

### Thematic coding

We first filtered all 114,257 tweets to identify those concerned with Covid-19. To do this, we searched for all tweets that included terms such as ‘coronavirus’, ‘COVID-19′, ‘pandemic’ and their variations. This identified 8249 Covid-19 related tweets across all stakeholders. Next, we randomly selected a sample of these and coded them manually to enable analysis of their content. This was an iterative process involving the identification of themes that emerged from the data and the development of a coding manual to inform subsequent (automated) coding. After coding about 15% of the tweets (N = 1237), it appeared that we had reached saturation in terms of the themes that emerged from the data, with each new tweet fitting one (or more) of the existing themes. We confirmed this by coding a further sample of tweets, ultimately manually coding a total of 1400 messages. As one of the aims of the paper was to inform understanding of the types of crime reported as being of concern during the pandemic, we manually coded the crime-related tweets according to the following categories: crime type (what type of crime a tweet focused on), modus operandi (information about how the crime discussed was perpetrated), vulnerability (information about behaviour that may make the public vulnerable to the modus operandi), and any advice offered (e.g. a phone number to report offences to, crime prevention advice, or links to Additional file 1).

Next, based on the most common themes and keywords that emerged from the qualitative analysis, we built a coding matrix to automate a content analysis of the tweets. This was implemented in Microsoft Excel. The coding matrix comprised a series of Boolean search terms that took the tweet text as input and generated dummy codes for a total of 45 themes as output. Here we note that, as this coding matrix and the Boolean terms were developed based on the emergent themes of our qualitative analysis, a different dataset of tweets (for example, from different stakeholders, or stakeholders from different countries) may require a modification of the themes, or the Boolean terms, considered for an automated content analysis inherent to the corpus of tweets in question. Table 1 provides examples of the Boolean terms used. In this case, those used to identify incidents of fraud and domestic abuse. As Table 1 shows, some of the Boolean terms were more extensive than others.

**Table 1:** Boolean terms used to code themes for fraud and domestic abuse

Theme	Boolean search term
Fraud	*CONTAINS*:[{"*fraud*","*scam*","*phish*","counterfeit","illegal","fake","pirate*", "forgery","forged", "falsified", "suspicious", "unexpected", " unsolicited"} *AND CONTAINS*: {"email*", "text*", "account*","call", "attachment*","link*","ad*"," good*", "website*", "tax", "photo*","message*"," impersonate "," pretend,*takefive ","actionfraud"}] *OR CONTAINS*: [{"*fraud*","*scam*"}]
Domestic Abuse	*CONTAINS*: {"domestic", "intimate","partner","home"} *AND CONTAINS *{"abuse", "violence"}

(* is a wildcard operator, such that ‘violen*’ would identify terms such as ‘violence’, ‘violent’ and so on)

To test the reliability of the approach, we applied these functions to another sample of 850 tweets (selected at random) that were not used to generate the keywords or identify themes in the data. Doing so generated new ‘dummy’ values for each tweet for each of the 45 themes discussed above. As an example, consider a tweet that warned that during the pandemic the selling of medical counterfeits on people’s doorsteps was increasing and that incidents could be reported to Action Fraud. For this sort of tweet, the Boolean logic would generate positive values for the tweet being: crime related, concerned with fraud, discussing an exploit that was an example of doorstep crime, that the crime involved Covid-19 relief products, and that advice was provided about who to report this kind of incident to and how. For all other ‘dummy’ variables, zero values would be recorded. To test the accuracy of the automated coding, we also manually coded these 850 tweets and computed a simple index of inter-rater reliability using Cohen’s Kappa statistic (Cohen 1960). The Cohen’s Kappa score (k = 0.87) calculated indicated near perfect agreement between the tweets that were manually- and those that were automatically-coded. However, where possible, we modified the original string search function to improve accuracy further. The automated coding was then applied to all 114,257 tweets.

It is important to note that as this is a qualitative analysis, the coded categories are not mutually exclusive (e.g. cybercrime and fraud); more than one type of crime could be discussed within a single tweet. To preserve the contextual richness of the data we coded tweets as concerning all the crime types to which they referred.

## Results

### Types of tweet

Table 2 shows the proportion of tweets that focused on crime or other issues for the period^6^ before and during the pandemic, as well as the proportion of tweets that focused specifically on Covid-19. For all stakeholders, it appears that for each period considered, the majority of tweets focused on non-crime issues, but that this was particularly the case for the Covid-19 period. Such Tweets focused on, for example, government guidance about public behaviour during the pandemic (e.g. regarding frequent handwashing, monitoring symptoms and self-isolating accordingly) and general policing (e.g. police community presence, traffic announcements, and so on). A similar pattern emerged when we focused on the Twitter accounts of the Territorial police forces only.

**Table 2:** Tweet types—crime context

	All stakeholders			Territorial Police forces		
	All tweets pre-Covid-19 era (1.09.2019–01.02.2020) (%)	All tweets Covid-19 era (01.02.202–10.05.2020) (%)	Covid-19 tweets (23.01.202–10.05.2020) (%)	All tweets pre-Covid-19 era (1.09.2019–01.02.2020) (%)	All tweets Covid-19 era (01.02.2020–10.05.2020) (%)	Covid-19 tweets (23.01.2020–10.05.2020) (%)
Non-crime context	68.4	73.9	76.7	64.5	73	75.5
Crime context	31.6	26.1	23.3	35.5	27	24.5
Total	57,741	56,516	8249	42,115	39,595	4729

Table 3 shows the proportion of tweets that were original messages, retweets, replies, or quotes. The figures shown are for all tweets, those that concerned Covid-19, those that concerned Covid-19 and Crime, and those that were sent by territorial police forces. It is apparent that the proportion of original tweets sent was about 50% of all tweets, regardless of whether they concerned Covid-19 or not. However, relative to other tweets, for those that concerned Covid-19, a much larger proportion of messages were retweets. This was true regardless of whether the Twitter account belonged to a territorial police force or another type of stakeholder. At least for this sample of Twitter accounts, (like the virus itself) it seems that messages about Covid-19 were more likely to spread than were other types of message.

**Table 3:** Tweet types

Tweet type:	All stakeholders			Territorial Police forces		
	All tweets (%)	Covid-19 tweets (%)	Covid-19 crime tweets (%)	All tweets (%)	Covid-19 tweets (%)	Covid-19 crime tweets (%)
Regular tweet	49.4	44.4	50.0	52.0	46.5	51.7
Retweet	26.8	45.7	43.2	23.1	40.7	41.8
Reply	19.1	6.2	2.6	20.1	8.1	2.2
Quote	4.7	3.7	4.2	4.8	4.7	4.3
Total	114,257	8,249	1917	81,710	4729	1160

The increased percentage of retweets that were Covid-19 related might be due to the urgency associated with spreading information regarding the pandemic, as retweets require only ‘one click’ to send, which is simpler than creating an original tweet.

### Are tweets contagious or are they self-isolating?

**Fig. 1:**
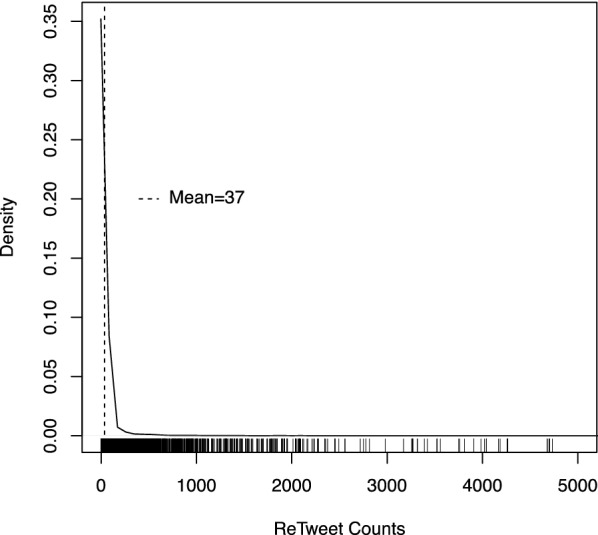
Reproductive ratio of Tweets

Whether a tweet is retweeted or not is considered crucial for the dissemination of information, and is an important measure of the impact of the intended message and the visibility of the tweeting account (Suh et al. 2010; Boyd et al. 2010; Hong et al. 2011; Zaman et al. 2010; Fernandez et al. 2017). The above descriptive statistics consider the proportion of tweets that were retweets, but not how frequently messages sent by the stakeholders were retweeted. We consider the latter here. For all tweets, we find that the mean number of retweets—regardless of who retweeted them—was 37 (median = 5). However, it is also evident from Fig. 1 that some tweets were more ‘viral’ than others. For example, nineteen percent of all tweets were never retweeted, sixty-six percent were retweeted less than 10 times, whilst five percent were retweeted more than 100 times (one percent more than 500 times).

Given that the rate at which messages are retweeted is considered an important indicator of their impact, this raises questions about whether there are particular characteristics of tweets that are associated with the frequency with which they are retweeted. Some of these might be considered when stakeholders post messages to try to increase the impact of tweets. Previous analyses of Twitter accounts (e.g. Suh 2010; Fernandez et al. 2017) have shown that characteristics of the account (e.g. the number of followers an account has), as well as the content of the tweet (e.g. whether it includes a URL) are significantly associated with the likelihood that a tweet will be retweeted. As far as we are aware, no studies have conducted this kind of analysis for police Twitter accounts during a pandemic (but for a general analysis of police Twitter accounts, see, Fernandez et al. 2017).

To examine this issue, we conducted a statistical analysis to examine which factors were associated with the frequency with which messages (original messages not those that were retweets of existing material) were retweeted. Given the skewed distribution of the data, and the fact that we have many zeros, we use a hurdle model to estimate the frequency with which tweets were retweeted. Hurdle models (e.g. Loeys et al. 2012; McDowell 2003) are used where two data-generating processes are assumed to contribute to the generation of zeros and non-zero values in a dataset. A logit model is used to estimate the probability of observing non-zero values, and an appropriate (truncated at zero) count model is used to estimate the likelihood of observing particular non-zero values (e.g. 1, 2, 3, 4, ….). In the case of the latter, we use a negative binomial model as this provided a much better fit to the data than did a Poisson model. This was illustrated by an improvement (of 1,650,778) in the Akaike Information Criterion (AIC), and the inspection of hanging rootagrams, which show the extent to which the model correctly predicts different counts of retweets (See Appendix A).

Zero-inflated negative Binomial (ZINB) models offer an alternative to the Hurdle model. ZINB models also estimate the influence of two data-generating processes, but do so using a slightly different approach; one part of the model estimates excess zeros, while the other models non-zero counts and non-excess zeros. That is, both parts of the model estimate zeros but different types of them (excess and non-excess). As discussed elsewhere (e.g. Loeys et al. 2012; McDowell 2003; Zeileis et al. 2008), the two types of model often yield similar results but the findings from the Hurdle models are easier to interpret. For this reason, we employ the latter here.

For this analysis, we included variables constructed by extracting data from the content of the tweets as well as the metadata associated with the accounts. For the latter, we considered the effect of the number of followers an account had, the number of times the account had ‘favourited’ other tweets (a measure of account activity), and whether messages were posted by a territorial police force. For the former, we considered whether the tweet text was about Covid-19, whether the message was about crime, whether messages were about crime in general (as opposed to a specific offence type), whether tweets quoted other tweets, whether tweets were a reply, and whether tweets included a photo. While some of these variables cannot be manipulated by an account holder, some of them quite clearly (e.g. the latter variables) can.

Analyses were conducted using the *hurdle()* function in the R *pscl* library. Table 4 shows the results. It is apparent that whether a tweet was retweeted, and the number of times it was retweeted, was positively associated with the number of followers an account has, the activity of the account as measured by the number tweets ‘favourited’ by the account owner, whether the tweet included reference to Covid-19, whether it covered a crime topic, and whether it included a photograph. For both parts of the model, the partial regression coefficients shown are exponentiated (i.e. they are odds ratios) and are consequently multiplicative. So, for example, if a tweet contained a photo, that message was almost twice as likely to be retweeted than a tweet that did not, all else equal. Replies, quoted tweets, and tweets that discussed crime in general (as opposed to specific crime types) appeared to be less likely (and less frequently) to be retweeted than did other types of messages. Tweets sent from territorial police force accounts were more likely to be retweeted than messages sent by other account holders, but when they were retweeted, they appear to have been retweeted less frequently. Having examined the likelihood that tweets would be retweeted, we looked at the content of the messages in more detail.

**Table 4:** Hurdle model exponentiated coefficients (odds ratios) for the frequency of retweets

	Logit		Negative Binomial	
	e^β^	Z-Score	e^β^	Z-Score
Followers (per 100 k)	1.09*	7.78	1.13*	54.95
Favourite Activity	1.22*	58.27	1.01*	74.60
Covid-19 topic	1.78*	7.27	1.56*	21.88
Crime topic	1.95*	9.54	1.21*	17.05
Territorial Police Force	1.26*	7.46	0.75*	− 24.09
General Crime topic	0.83**	− 2.89	0.74*	− 13.67
Includes Photo	1.96*	20.63	1.35*	24.01
Quotes	0.58*	− 10.66	0.93**	− 2.96
Replies	0.06*	− 88.01	0.38*	− 46.71

*p < 0.00001, **p < 0.01

### Types of crime

**Fig. 2:**
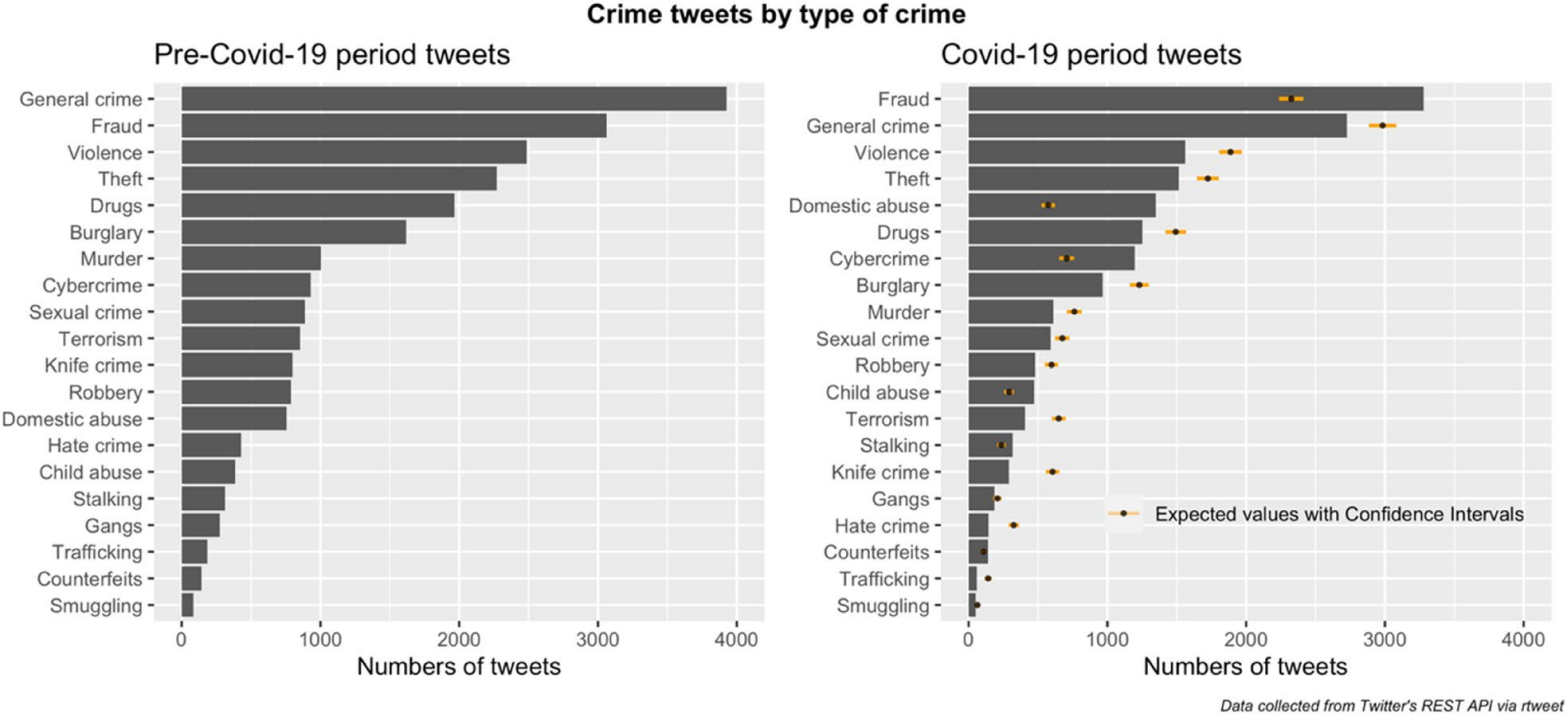
Crime tweets by type of crime

Figure 2 shows the results of a content analysis concerning the crime type themes discussed in tweets posted during the pre-Covid-19 period (n = 19,790) and Covid-19 periods (n = 14,779). Here, we focus only on the crime related (subset of) tweets as one of the aims of our study was to assess the crime trends being reported by the police forces on their twitter accounts before and during the pandemic. There were clear differences in the frequency with which tweets concerned the different types of crime. And, while there was an association between which crime types received most coverage across the two periods, there were differences. To highlight these, for the Covid-19 period, we also estimate the expected values (and 95% confidence intervals), assuming that the proportion concerned with a particular crime theme during this period would be the same as that for the pre-Covid-19 period.^7^ Relative to the Pre-Covid-19 period, for the Covid-19 period we see higher than expected frequencies of tweets concerned with fraud, domestic abuse, cybercrime, child abuse and stalking, and drops in (for example) those concerned with general crime, violence, burglary, terrorism and knife crime.

**Fig. 3:**
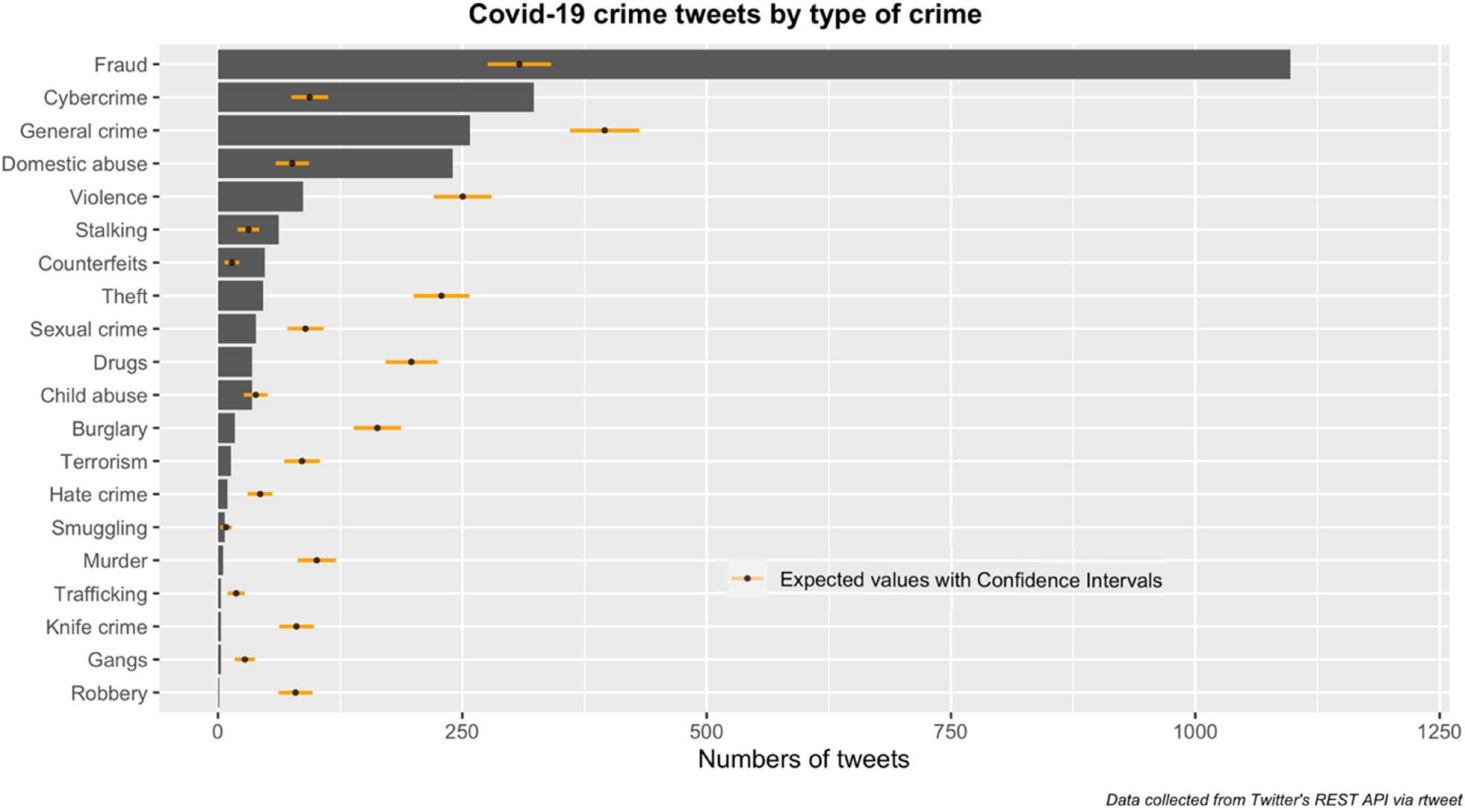
Covid-19 crime tweets by type of crime

Looking closely at the Covid-19 themed crime tweets in particular (Fig. 3), the majority concerned fraud (57.22%), followed by cybercrime (16.85%), general crime (13.46%) and domestic abuse (12.52%). For this paper, we subsequently concentrate on these four crime themes.

Table 5 provides example tweets (reported verbatim) for each crime type to illustrate the kinds of issues covered. As noted above, in some cases (e.g. example 1 for fraud), tweets may refer to two of the crime themes that emerged in our content analysis (in this example, cybercrime and fraud).

**Table 5:** Tweet examples (reported verbatim)

	Example 1	Example 2	Example 3	Example 4
Fraud	#Scam—Fraudsters exploiting spread of #COVID19 #coronavirus for #fraud & #cybercrime—Victim losses from 21 reports since Feb 2020 total over £800 K. 10 reports of victims trying to purchase face masks from fraudulent sellers Read for details & advice (link)	More people may fall victim to #onlineshopping fraud as they self-isolate due to #COVID19 You are a victim of online shopping fraud if you buy goods from an online seller that never arrive. Find out how to protect yourself(link)	Be careful of scams as #coronavirus continues to spread. Visit Action Fraud for more advice and information ⬇(link)	Stamp out #COVID19 Doorstep scams. Learn the signs to spot or speak up for those that may have fallen victim. Tell the charity @CrimestoppersUK what you know 100% anonymously—0800 555 111 or online: (link)
Cybercrime	We've been working tirelessly alongside our colleagues at @CityPoliceFraud to investigate alleged frauds linked to #COVID19. We will proactively pursue people exploiting this national crisis for personal profit. Report fraud/cyber crime to @ActionFraudUK (link)	Cyber criminals are exploiting peoples interest in COVID-19 to steal login details & download malware. This has increased as more people have become reliant on internet based services because they are working from home or in isolation	Cyber Criminals are using #CoronaVirus to target people with #Ransomware. Techniques seen since the start of #Covid19 include emails with links claiming to have important updates, which once clicked lead devices to being attacked (link)	During the #covid19 crisis we are continuing to pursue offenders engaging in the sexual abuse of children online, and we will be sharing lots of resources to help children, parents & carers improve their #OnlineSafetyAtHome. More at (link)
General crime	RT@INTERPOL_HQ : Police around the world are working to support government measures and counter new crimes linked to #COVID19. (link)	RT@SouthCovWMP: Covid 19 Update, Response officers are on Duty 24/7 protecting the most vulnerable, NHT teams are patroling local ward areas communicating the new covid 19 regulations, fcid teams are investigating crimes supporting victims of crimes. (link)	Recorded crime has fallen during Scotland’s response to the coronavirus according to our early indications. Find out more: (link)	Be vigilant against criminals using the publicity around coronavirus as a chance to target the vulnerable—(link)
Domestic abuse	"If you suspect your neighbour is experiencing domestic abuse, you can call the domestic abuse advocacy service on 0300 790 6772 for advice. if you suspect someone’s life is in danger, call 999.\r\n covid19 (link)	Staying home should not mean at risk. If you are a victim of #DomesticAbuse—remember #YouAreNotAlone. What happens when you report domestic violence to us during the #COVID19 outbreak? Watch our short video here: (link) We are still here and we can help	With #COVID19 restrictions, people are now isolating within their homes. This means that we may see a rise of domestic violence and child abuse cases. If you're in an emergency situation, but can’t talk, here's how to let us know you need help: (link)	People facing violence/controlling behaviour at home should still report their experiences to police or seek advice & support from local domestic abuse services. Officers will attend calls for help and arrest perpetrators despite the additional pressures on the service. #Covid19

In terms of how the crimes discussed were perpetrated, the majority of Covid-19 fraud tweets concerned tax matters, Covid-19 relief materials and scams associated with working from home. Covid-19 cybercrime tweets also tended to focus on offences related to Covid-19 relief or working from home. Covid-19 general crime tweets mostly included warnings about criminals exploiting the pandemic (in general terms) and victimisation. Tweets concerned with domestic abuse tended to concentrate on the impact of the lockdown (i.e. changes to mobility and time spent at home) on this form of offending. Most of the tweets that covered these crime specific themes also offered some form of advice on how to avoid victimisation, or web links where readers could find further information on the topic (via a URL link embedded within the tweet to an external source of information). However, very rarely were details provided (within a tweet) about how victims could report offences. For example, for the Covid-19 tweets, for only 3.1% of those concerned with fraud (n = 1,097), 6.5% of those concerned with cybercrime (n = 323), 16.3% of those concerned with general crime (n = 258), and 26.7% of those concerned with domestic abuse (n = 240) was a reporting number provided.

**Fig. 4:**
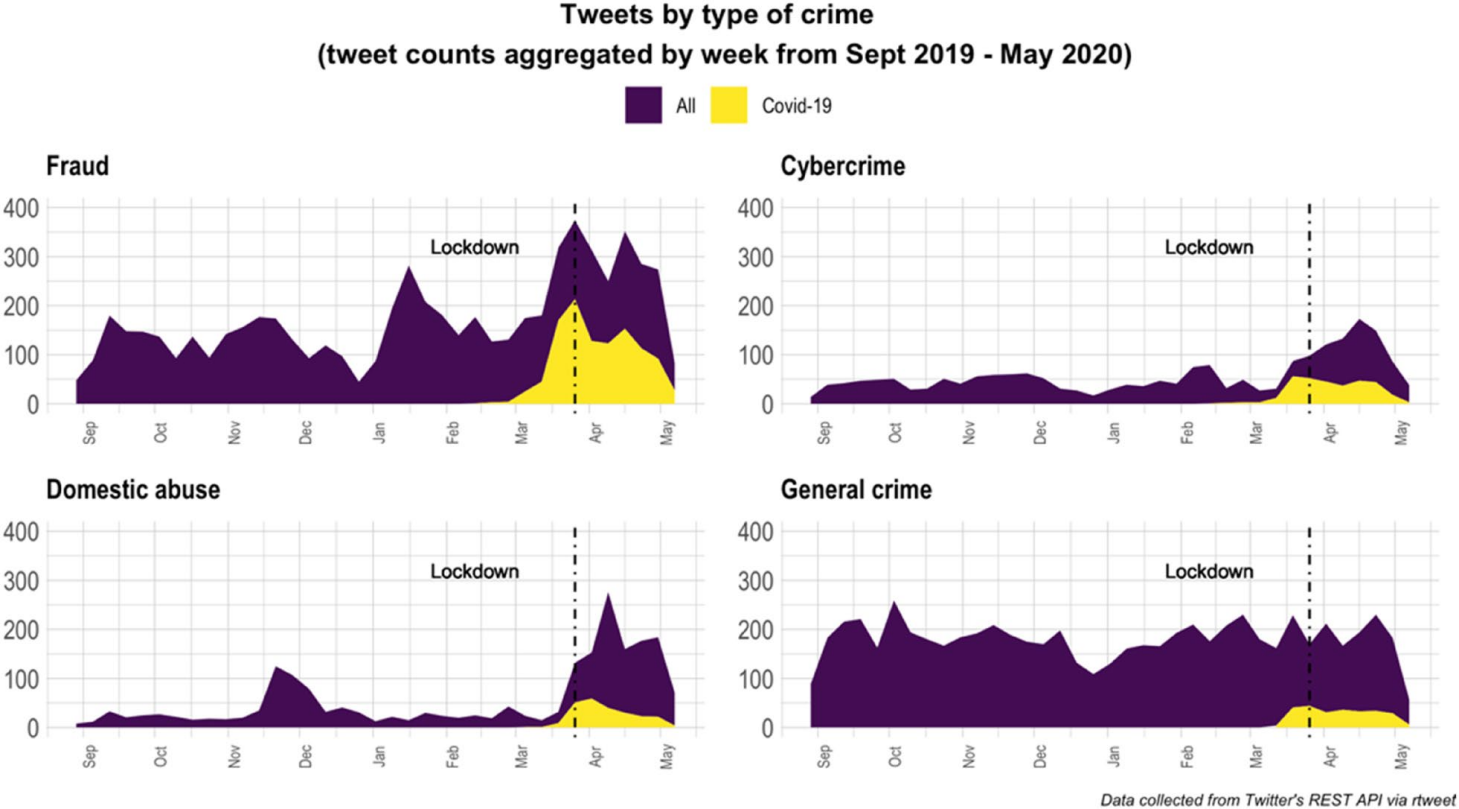
Frequency of Fraud, Cybercrime, Domestic abuse and General crime tweets

Next, we consider changes in the pattern of tweets over the course of the pandemic. Figure 4 shows weekly time series data regarding the frequency of tweets concerned with fraud, cybercrime, domestic abuse and general crime. While the frequency of tweets concerned with non-specific crime matters (ie. general crime and offending) remained relatively steady throughout the entire period considered, there was an increase in tweets concerned with fraud, domestic abuse and cybercrime from March 2020 onwards. Initially, these tweets explicitly referenced the pandemic (see the frequency of Covid-19 tweets), but the frequency with which this was the case appeared to decline over time.

## Discussion and conclusions

The aim of this paper was to analyse the content of tweets posted by UK law enforcement and associated agencies during a time of global disruption. In this case, the disruption was due to the Covid-19 pandemic, but the findings of the research also have implications for handling other disruptions and the use of social media by law enforcement stakeholders more generally. The analysis of 114,257 tweets and their metadata indicate that (a) most of the tweets focused on issues that were not specifically about crime; (b) during the time of crisis the stakeholders in question tended to increase their retweeting activity rather than creating original tweets; (c) the visibility of an account (number of followers and favouriting habits) and the richness of the content (discussing Covid-19, crime specific issues and including media such as images) were associated with the likelihood of messages spreading (both in terms of whether they were retweeted and the frequency with which this was so); (d) relative to the preceding 5 months, during the first 5 months of the pandemic tweets on Fraud, Cybercrime and Domestic abuse increased significantly.

Our finding that most tweets were not crime-focused, but centred instead on encouraging the public to comply with government guidance about behaviour during the pandemic or concerned general policing, is broadly in line with Walsh’s 2019 study on the tweeting practices of migration policing actors, which found that 79.5 per cent of tweets sent by policing agencies were informational and intended to raise awareness about policing and operational activities and capacity. In our case, this was even more so when we considered the Covid-19 tweets. It seems that the stakeholders from our sample were ‘lending’ their tweeting capacity to spread public health-oriented information to raise awareness about the pandemic and its prevention. While the pandemic has proven to be a call for ‘all hands-on deck’, straying from a crime reduction focus may prove counterproductive in some respects. For example, as noted, and in agreement with previous studies (e.g. Fernandez et al. 2017, Heverin and Zach 2010, Velde et al. 2015), users tend to retweet law enforcement tweets that contain crime specific content, and are content-rich with media such as photo, video, and URL’s. Other research also suggests that users favour retweeting messages that contain time-sensitive material (Boyd et al. 2010), which may be particularly relevant in times of crisis. Therefore, while it is crucial to spread the message about the ‘general picture’ and urgent issues connected to the disruption in question (in this case, social distancing and lockdown measures), law enforcement stakeholders should consider whether it is better to maintain a focus on the dissemination of crime-specific prevention tweets that are within their mandate. Stakeholders should also consider prioritizing information (and in doing so boost its impact) that is time-sensitive, and ensure that as well as discussing such content, they use adjectives to convey its urgency, such as ‘urgent’, or phrases like ‘time-sensitive’ (as perhaps they would in an email). Such (in fact all) messaging would need special care to check the validity of the content prior to dissemination, as urgency messaging can be fertile soil for spreading fake news or misinformation. Moreover, care would need to be taken to not overuse such phrasing, which would likely dilute its potency.

The detected increase of tweets on fraud, cybercrime and domestic abuse is in line with preliminary reports of these crimes being on the rise during the pandemic. The surge in the frequency of (all) tweets concerned with Fraud (Fig. 4) is clearly also explained by the occurrence of Covid-19 specific tweets that mention this type of crime. While there is some evidence of a similar pattern for cybercrime and domestic abuse, this is less clear—tweets concerned with these crime types remain elevated throughout the Covid-19 period, but those that explicitly mention Covid-19 account for a much smaller fraction.

One reason for this could be that the particular modus operandi employed to commit these types of crimes may not have changed due to Covid-19, even though the opportunity or motivation to commit them did. For example, with more people staying at home, the opportunity for domestic abuse may increase. Likewise, with more people staying at home and using the internet to work remotely, the risk (per unit of calendar time) of cybercrime would be increased. These are indirect effects of the virus. In contrast, fraudsters have been adapting their modus operandi to create and exploit specific opportunities that the restrictions associated with Covid-19 presents. For example, fraudsters have been selling fake coronavirus testing kits or impersonating relevant coronavirus crisis response governmental bodies to defraud people. At the same time, the fact that people may be increasingly vulnerable to fraud and cybercrime during the pandemic may be explained by how we react when we feel threatened, scared and exposed to uncertainty. For example, experimental research on Protection Motivation theory (PMT: Rogers, 1975)—which considers how people view suggested actions when they perceive a threat—suggests that when people perceive a high expectation of threat exposure, they are easier to persuade using any information that offers a possibility of threat evasion. Moreover, research by Floyd et al. (2000) suggests that fear-stimulating communications increase the adoption of proposed adaptive behaviours. These findings have informed a number of ‘public health’-type programmes intended (for example) to encourage smoking cessation (Greening 1997) or to promote cyber secure behaviours (Vance et al. 2012); PMT was also recently used to encourage social distancing and protective measures for hospital staff against the virus (Kemp 2020; Barati et al. 2020). However, in the case of fraud, it may be that criminals are exploiting the fear associated with the pandemic and the consistent messaging about the need for positive protective action. This may create the conditions for them to trick members of the public into paying for counterfeit (or non-existent) goods (e.g. a vaccine, testing kits, protective equipment and so on) or services (e.g. tax relief schemes). This is an unintended consequence of well-intended messaging. To counter this, our recommendation would be that stakeholders should be mindful when sharing information that may trigger hyper-defensive behaviour and—where possible—provide clear advice, recommendations, or links to trusted sources that can do so; recall that only 3.1% of the tweets we analysed provided a reporting number within the Covid-19 fraud tweets.

Another point worth noting concerns the precise timing of tweets in relation to that of the lockdown. In all cases, some Covid-19 tweets concerned with crime started to be posted prior to the lockdown. However, for Covid-19 related fraud, cybercrime and crime in general, Twitter activity commenced sooner and increased more rapidly than it did for domestic abuse. In the case of domestic abuse, the peak in Twitter activity observed was several weeks after the lockdown had started. Given the potential for the lockdown to make this crime more likely, and because victims/survivors may be less able to report offences under such conditions (as they may be more closely monitored by offenders) this is unfortunate. It is easy to say this in hindsight, but it would have been better to communicate about this type of offending when there was more opportunity for victims to contact support services and for their support networks to be able to meet or contact them.

For the avoidance of doubt, the above is not a criticism of the communication strategies of the LEAs examined here, as the conditions are unprecedented and there was much uncertainty about the government’s strategy, including the timing of the lockdown. However, lessons should be learned. With respect to future communications strategies, it would be sensible for agencies to engage in short-term foresight activities to review which crimes are most likely to be affected by a disruption (such as a pandemic) and, for which crimes the window of opportunity to do something about the problem is collapsing most quickly. Most of the guides for use of social media by police (at least, those available to the public) emphasise the need for freedom of information and advice on privacy and confidentiality best practices (see for example, Guidelines On The Safe Use Of The Internet And Social Media By Ministry of Defence Police Officers^8^). Or, as discussed in Fernandez et al. (2017), they provide general engagement guidelines, such as the need to use simple language and clear and focused messaging.

However, to get the most out of it, social media mobilisation in times of disruption may require a more systematic and strategic approach. As discussed, the crimes about which information is to be disseminated could be prioritised according to the emergent, or anticipated disruption scenario. But LEAs may also wish to consider adopting a more coordinated and structured approach. For example, Ekblom (2011) suggests mobilisation involves at least seven tasks, encapsulated by the acronym CLAIMED:

*Clarify* the specific crime prevention roles, responsibilities and tasks that need to be undertaken in relation to a given crime problem (in the present case, to address the crime risks associated with COVID-19); or the inadvertent crime promotion actions that should be ceased (e.g. insecure procedures for tracking and tracing that provide opportunities for fraudsters or distraction burglars).

*Locate* the individuals and organisations best-placed as dutyholders or wider stakeholders to undertake these roles, in terms of, say, expertise, local knowledge, legitimacy, coverage on the ground; and having achieved these steps,

*Alert* them about the existence and scale of the problem,

*Inform* them about the nature of the problem, what the causes and consequences are, who are the offenders etc.,

*Motivate* them e.g. by incentives, regulations and laws, naming and shaming, ‘the right thing to do’,

*Empower* them with appropriate know-how, legal powers, tools, funds and so forth; and if appropriate,

*Direct* them through audits, commitment to objectives, performance standards and so on.

While this framework was initially developed for thinking about crime reduction actors (e.g. place managers), in times of disruption the above elements of the framework will apply to the public too. Note also that while some of the tasks, roles and responsibilities in question will be direct preventive interventions intended to reduce crime opportunities (e.g. how to avoid succumbing to COVID-19 related fraud), others will relate to supporting activities (e.g. providing training for interventions) or disseminating influence further down the chain or to other stakeholders. In a similar vein, Fielding and Caddick (n.d) suggest that there are six communication purposes associated with police use of social media, to: Publicise, Advise, Inform, Warn, Appeal and Engage.

Such frameworks could be used at operational or strategic levels, both to construct individual tweets (‘have we considered motivation?’ etc.) and to coordinate Twitter campaigns (e.g. does the messaging have clear implications for all relevant stakeholders?). They could thus be used to think systematically about what messages are intended to achieve and to subsequently tailor the messages to address these goals. More broadly, the sending and receiving of tweets can be considered from a ‘system of influence’ perspective. As an illustration to the Covid-19 scenario and applying the CLAIMED framework, an INFORM tweet containing information that wearing masks is now mandatory, should contain an ALERT that this can also be exploited by criminals through personal protective equipment scams. On the basis of our regression analysis, stakeholders could additionally boost the impact of ALERT tweets by uploading a photo or other media that is relevant to the ALERT. Conscious of the 280-character limit, law enforcement actors may wish to consider adding such information through the ‘thread’ creation option, which allows the insertion of additional tweets as an attachment.

Of course, this study is not without limitations. Chief amongst these is the fact that our findings are for a sample of UK police organisations. Different findings may be observed for police organisations in other countries, or for other UK stakeholders not sampled here, or the personal accounts of police officers in these jurisdictions. However, while extending the sample would be beneficial, we believe that the insights provided here achieve our intended aims. Nevertheless, creating an open source cohesive repository of all verified twitter accounts by UK police forces would be highly beneficial, for users, as well as for research.

In closing, we emphasise three points. The first is that the pandemic has made it very clear to all that we live in a changing world. Additional waves of the pandemic may lead to further changes. However, the pandemic is only one dimension of change. For example, changes to technology—to include rapid advances in artificial intelligence (e.g. Caldwell et al. 2020), internet connectivity (e.g. Blythe and Johnson 2020) and biotechnology (Elgabry et al. 2020)—, society (e.g. Brexit) and the environment (e.g. climate change) all have potential implications for crime that require attention (Johnson et al. 2018; Topalli and Nikolovska 2020). For example, like the pandemic, they have the potential to create uncertainty or changes to people’s routine activities that criminals might exploit. Doing something about these, including communicating about the potential impact of these changes on crime, will be important, and stakeholders and researchers should now be thinking about when and how best to do this. Not doing so may mean missing windows of opportunity. The second point is that there currently exists relatively little research on the use of Twitter by law enforcement agencies (for an exception, see Fernandez et al. 2017). Current guidance tends to focus on the composition of messages, but the focus is on compliance with regulation and avoidance of (say) reputational damage rather than a consideration of what is effective in terms of reducing crime or encouraging crime reduction activity. As such, we encourage other researchers to look at police use of Twitter with a view to developing a literature on ‘what works’. Finally, in the current study, we observed increases in Twitter activity about particular forms of crime (fraud, cybercrime and domestic abuse) during the pandemic. Future work might examine the extent to which Twitter data serves as an open source ‘leading indicator’ that anticipates, in real-time, changes to crime problems of this nature.

### Supplementary information

**Additional file 1**. Stakeholder list.

## Data Availability

Not applicable.

## Appendix A: Hanging rootograms for poission and negative binomial hurdle models

Hanging Rootograms were generated using the *rootogram()* command in the R *countreg* library. As shown by the figure on the right, the Poisson Hurdle model dramatically underpredicts counts up to 6 and overpredicts those between 8 to (about) 30. For the negative binomial Hurdle model, the fit is clearly much better.

## Footnotes

1 See: https://www.ucl.ac.uk/jill-dando-institute/research/covid-19-special-papers; https://covid19-crime.com/; https://covid19.counciloncj.org/.

2 https://upsi.org.uk/oscar.

3 Action Fraud is the UK’s national reporting centre for Fraud and cybercrime (see https://www.actionfraud.police.uk/what-is-action-fraud).

4 For example, the primary twitter account of the Greater Manchester police is “@gmpolice”. However, this police force also has specialized verified accounts such as “@GMPCityCentre” which concentrates on the policing of Manchester city centre, “@gmpfraud” which concentrates on providing updates from the Greater Manchester Police Economic Crime Unit, “@gmptraffic” which concentrates on providing updates on Greater Manchester traffic, and so on.

5 A peer-reviewed R language package designed for implementation of calls to collect and organize Twitter data via Twitter's REST and stream Application Program Interfaces (API)—can be found on https://developer.twitter.com/en/docs.

6 **Pre-Covid-19 era defined by the period for which by* complete data were available for all stakeholders—01.09.2019 and first Covid-19 case recorded in the UK—31.01.2020; *** Covid-19 era determined by first case recorded in the UK*—31.01.2020 and ease of Lockdown initiation by the Government—10.05.2020;****Covid-19 tweets era* determined by the first tweet on Covid-19 from our stakeholders—23.01.2020 and ease of Lockdown initiation by the Government—10.05.2020.

7 The standard errors were then calculated using the standard formula, se. = sqrt[p(1-p)/n], where p is the proportion concerned and n is the sample size.

8 https://assets.publishing.service.gov.uk/government/uploads/system/uploads/attachment_data/file/329509/Guidelines-socialmedia-v1-jan13.pdf. Last accessed 17 September 2020.
